# Matching-adjusted indirect comparisons of PARP inhibitor combinations in metastatic castration-resistant prostate cancer across key populations

**DOI:** 10.1093/oncolo/oyag143

**Published:** 2026-04-16

**Authors:** Elena Castro, Di Wang, Stefanie Paganelli, Anja Haltner, Neo Su, Melissa Kirker, Jane Chang, Imtiaz A Samjoo

**Affiliations:** Department of Medical Oncology, i+12 Biomedical Research Institute.Hospital Universitario 12 de Octubre, Madrid, Spain; EVERSANA™, Burlington, Ontario, L7N 3H8, Canada; EVERSANA™, Burlington, Ontario, L7N 3H8, Canada; EVERSANA™, New York, NY, 10018, United States; Pfizer, Inc., New York, NY, 10001, United States; Pfizer, Inc., New York, NY, 10001, United States; Pfizer, Inc., New York, NY, 10001, United States; EVERSANA™, Burlington, Ontario, L7N 3H8, Canada

**Keywords:** metastatic castration-resistant prostate cancer, matching-adjusted indirect comparison, PARP inhibitor, talazoparib, homologous recombination repair, comparative efficacy

## Abstract

**Background:**

Without head-to-head trials comparing talazoparib plus enzalutamide (TALA+ENZA), olaparib plus abiraterone acetate and prednisone (OLAP+AAP), and niraparib plus abiraterone acetate and prednisone (NIRA+AAP) as first-line treatments for metastatic castration-resistant prostate cancer (mCRPC), treatment selection remains challenging. This study estimated the relative efficacy of TALA+ENZA vs OLAP+AAP and NIRA+AAP in unselected, homologous recombination repair (HRR)-deficient, and *BRCA*-mutated (*BRCA*m) populations.

**Methods:**

Unanchored matching-adjusted indirect comparisons (MAICs) were conducted using individual patient data from TALAPRO-2 (TALA+ENZA) and published summary-level data from PROpel (OLAP+AAP) and MAGNITUDE (NIRA+AAP). TALAPRO-2 patients meeting PROpel/MAGNITUDE eligibility criteria were included; remaining patients were reweighted to align on key baseline characteristics. Hazard ratios (HRs) and 95% confidence intervals (CIs) were calculated for radiographic progression-free survival (rPFS) and overall survival (OS).

**Results:**

In unselected patients, TALA+ENZA significantly prolonged rPFS vs OLAP+AAP (HR: 0.747; 95% CI, 0.583, 0.957), with no OS difference (HR: 0.821; 95% CI, 0.649, 1.039). In HRR-deficient patients, TALA+ENZA significantly prolonged rPFS vs OLAP+AAP (HR: 0.648; 95% CI, 0.423, 0.992), with no OS difference (HR: 0.834; 95% CI, 0.569, 1.223). Comparisons with OLAP+AAP in *BRCA*m were infeasible. Compared with NIRA+AAP, TALA+ENZA significantly prolonged rPFS and OS in HRR-deficient (HR: 0.406; 95% CI, 0.251, 0.655; HR: 0.554; 95% CI, 0.340, 0.902) and *BRCA*m patients (HR: 0.394; 95% CI, 0.222, 0.698; HR: 0.472; 95% CI, 0.247, 0.902).

**Conclusions:**

MAICs showed improved clinical benefit with TALA+ENZA vs OLAP+AAP and NIRA+AAP across multiple mCRPC populations and endpoints. Despite limitations of indirect comparisons, findings support TALA+ENZA as a first-line treatment option for mCRPC.

Implications for Practice Choosing the best first treatment for advanced prostate cancer is challenging because few clinical trials directly compare available options. This study applied a statistical method to evaluate three combination therapies that include poly (ADP-ribose) polymerase (PARP) inhibitors, modern drugs that represent a contemporary treatment strategy for patients with certain genetic mutations. Findings suggest that talazoparib plus enzalutamide may offer greater benefit than other combinations for patients with and without genetic mutations. These results can help guide treatment decisions and improve outcomes where direct comparative trial data are lacking.

## Introduction

Metastatic castration-resistant prostate cancer (mCRPC) is an advanced and life-threatening stage of prostate cancer, characterized by disease progression despite androgen deprivation therapy. Although recent advances in systemic therapies have modestly improved outcomes, long-term survival remains poor, underscoring the need for more effective first-line treatment strategies.^1^

Poly (ADP-ribose) polymerase inhibitors (PARPis) have emerged as a promising therapeutic class, particularly in patients with tumors harboring homologous recombination repair (HRR) gene alterations.^1^^,^^2^ In addition to their role as monotherapies for HRR-deficient mCRPC, PARPis are increasingly being explored in combination with androgen receptor pathway inhibitors (ARPIs) to potentially enhance antitumor activity in both unselected and HRR-deficient populations. Preclinical and clinical data support a synergistic interaction between these agents, driven by crosstalk between androgen receptor signaling and DNA damage repair pathways, even in the absence of HRR mutations.^3^^,^^4^

Three pivotal Phase 3 randomized trials have evaluated PARPi-ARPI combinations vs placebo-ARPI in the first-line mCRPC setting. TALAPRO-2 assessed talazoparib plus enzalutamide (TALA+ENZA) in both unselected (Cohort 1) and HRR-deficient (Cohort 2) populations^5^; PROpel evaluated olaparib plus abiraterone acetate and prednisone (OLAP+AAP) in an unselected population, with subgroup analyses in HRR-deficient and *BRCA*-mutated (*BRCA*m) patients^6^; and MAGNITUDE enrolled only patients with HRR gene alterations, including *BRCA* mutations, to assess niraparib plus abiraterone acetate and prednisone (NIRA+AAP); although the trial initially included an unselected cohort, that arm was discontinued early due to futility.^7^ These trials demonstrated favorable efficacy outcomes for PARPi-ARPI combinations in patients with asymptomatic or mildly symptomatic mCRPC and have led to regulatory approvals that vary by region and biomarker status. TALA+ENZA is approved by the United States Food and Drug Administration (FDA) for the treatment of patients with HRR-deficient mCRPC, and by the European Medicines Agency (EMA) for the treatment of patients with mCRPC in whom chemotherapy is not clinically indicated, regardless of HRR alteration status.^8^^,^^9^ OLAP+AAP is approved by the FDA for patients with deleterious or suspected deleterious *BRCA*m mCRPC, while the EMA has approved it for patients with mCRPC in whom chemotherapy is not clinically indicated, regardless of HRR alteration status.^10^^,^^11^ NIRA+AAP is approved by both the FDA and EMA for adult patients with deleterious or suspected deleterious *BRCA*m mCRPC, with the EMA additionally specifying use in patients for whom chemotherapy is not clinically indicated.^12^^,^^13^

These PARPi-ARPI combinations have not been evaluated in head-to-head trials, leaving a gap in direct comparative evidence. In the absence of such data, indirect treatment comparisons (ITCs) can offer valuable insights into relative efficacy. Matching-adjusted indirect comparisons (MAICs) are increasingly used to improve the validity of cross-trial comparisons by using individual patient data (IPD) to adjust for differences in baseline characteristics between studies. This reweighting process helps align populations across trials, accounting for known prognostic factors and treatment effect modifiers, thereby reducing bias in comparative estimates. Supplementary Appendix A  Figure S.1 provides an overview of MAIC methodology. MAICs can help bridge evidence gaps when direct comparisons are unavailable, supporting more informed treatment decisions. While not a substitute for randomized head-to-head trials, they are particularly useful in rapidly evolving therapeutic landscapes such as mCRPC, where multiple novel regimens have recently been approved based on separate pivotal studies. Notably, MAICs using IPD from the first TALAPRO-2 data cut have previously compared TALA+ENZA with other PARPi-ARPI combinations, demonstrating favorable efficacy outcomes.^14^ Building on these findings, we conducted new MAICs using the final TALAPRO-2 data cut to provide updated comparative efficacy estimates.

In this study, we used IPD from the final data cut of the TALAPRO-2 trial to conduct unanchored MAICs comparing TALA+ENZA with OLAP+AAP (using summary-level data from PROpel) and NIRA+AAP (using summary-level data from MAGNITUDE). Analyses were performed across three clinically relevant populations: unselected, HRR-deficient, and *BRCA*-mutated. The primary objective was to estimate the relative efficacy of these regimens in the first-line treatment of mCRPC, focusing on radiographic progression-free survival (rPFS) and overall survival (OS).

## Materials and methods

### Systematic literature search

A systematic literature review (SLR) was conducted in accordance with a pre-specified protocol (PROSPERO: CRD42021283512) and in adherence with established methodological and reporting guidelines for systematic reviews.^15–18^ The SLR identified randomized controlled trials (RCTs) evaluating first-line treatments in adult men (≥18 years) with asymptomatic or mildly symptomatic mCRPC. The initial search was conducted in September 2021 and most recently updated in August 2024. Full details of the SLR methodology including the eligibility criteria (Table S.1), sources searched (Table S.2), list of excluded studies (Table S.3), and the PRISMA flow diagram (Figure S.2) are provided in Supplementary Appendix B and have been published previously.^14^^,^^19^ For the current analysis, only pivotal RCTs evaluating the regimens of interest (ie, TALA+ENZA, OLAP+AAP, and NIRA+AAP) were included. Study quality was assessed using the National Institute for Health and Care Excellence (NICE) Single Technology Appraisal (STA) Evidence Submission Checklist.^20^

**Table 1.** Summary of included trials.

Trial; NCT	Phase Setting Blinding	Treatment arm (Experimental)	Treatment Arm (Control)	Population	*n* (Experimental)	*n* (Control)	Enrollment period Study sites and locations
**TALAPRO-2; NCT03395197^5^^,^^43^**	Phase 3 Multicenter Double-blind	Talazoparib 0.5 mg QD + Enzalutamide 160 mg QD	Placebo QD + Enzalutamide 160 mg QD	Unselected	402	403	January 2019-September 2020 200 centres across 26 countries covering North America, Europe, Middle East, South America, South Africa, Asia-Pacific
				HRR-deficient	200	199	December 2018-January 2022 142 centres across 26 countries covering North America, Europe, Middle East, South America, South Africa, Asia-Pacific
				*BRCA*m	71	84	
**PROpel^a^; NCT03732820^21^**	Phase 3 Multicenter Double-blind	Olaparib 300 mg BID + Abiraterone acetate 1000 mg QD + Prednisone/Prednisolone	Placebo BID + Abiraterone acetate 1000 mg QD + Prednisone/Prednisolone	Unselected	399	397	October 2018-March 2020 126 centres across 17 countries covering North America, Europe, Asia, and South America
				HRR-deficient	111	110	
				*BRCA*m	84	83	
**MAGNITUDE^b^; NCT03748641^7^**	Phase 3 Multicenter Double-blind	Niraparib 200 mg QD + Abiraterone acetate 1000 mg QD + Prednisone/Prednisolone	Placebo QD + Abiraterone acetate 1000 mg QD + Prednisone/Prednisolone	HRR-deficient	212	211	May 2019-March 2021 318 centres across 28 countries covering North America, Europe, Asia-Pacific, South America, Middle East, Africa
				*BRCA*m	113	112	

Abbreviations: AAP, abiraterone acetate plus prednisone/prednisolone; BID, twice daily; BPI-SF, Brief Pain Inventory-Short Form; *BRCA*m, *BRCA*-mutated; HRR, homologous recombination repair; mCRPC, metastatic castration-resistant prostate cancer; mg, milligram; N, number of patients; QD, once daily.

^a^In the PROpel trial, 25.8% of patients in the olaparib + AAP arm and 20.2% in the placebo + AAP arm were symptomatic in the unselected population (symptomatic defined as BPI-SF score ≥4 and/or opiate use).

^b^In the MAGNITUDE trial, 23.6% of patients in the niraparib + AAP arm and 22.7% in the placebo + AAP arm in the HRR-deficient population had received ≤4 months of AAP therapy for first-line mCRPC.

**Table 2.** Baseline characteristics of included trials.

Trial; NCT	Weighted average of active treatment and control arms												
	Age (mean years)	Caucasian (%)	ECOG PS 0-1 (%)	Gleason score ≥8 (%)	Baseline PSA (median ng/mL)	Time since initial diagnosis (median years)	BPI-SF ≤3 (%)	Bone metastases (%)	Proportion with visceral metastases				
									Total	Lung or Liver	Node	Liver (%)	Lung (%)
**TALAPRO-2; NCT03395197^a^**	70.6	61.9	100	70.1	17.18	2.84	99.4	83.8	NR	15.7%	39%	3.5	13.2
**PROpel; NCT03732820^21^**	69.5^b^	70	99.8^c^	65.7	17.36^b^	3.05	74.6	86.5	NR	NR	31.7%^d^	4.1	10.3
**MAGNITUDE; NCT03748641^7^**	69	74%	100	67.9^e,f^	19.40	NR	91	83	21.3%	NR	NR	7.4	10.6

Abbreviations: BPI-SF, Brief Pain Inventory-Short Form; ECOG, Eastern Cooperative Oncology Group; ng/mL, nanogram/milliliter; NR, not reported; PS, performance status; PSA, prostate-specific antigen.

^a^Based on the clinical study report of Cohort 1 provided by Pfizer reporting results at the data cutoff date of March 28, 2023.

^b^Median value was reported.

^c^Data missing in 0.3% of patients in each treatment arm.

^d^PROpel reported distant and locoregional lymph node metastases. The proportion of participants with distant lymph node metastases and locoregional lymph node metastases was 32% and 21%, respectively.

^e^Based on the full-text publication reporting results at the first interim analysis (data cutoff date of October 8, 2021).^44^

^f^Characteristics reported at diagnosis.

### Feasibility assessment

A qualitative feasibility assessment was conducted to ensure that the studies of interest were sufficiently similar for performing MAICs. This assessment included a review of trial design, eligibility criteria, baseline patient characteristics, outcome availability and definitions, the nature of interventions and comparators, and the availability of IPD.

### Outcomes and data sources

This analysis evaluated two key efficacy endpoints: rPFS as assessed by blinded independent central review (BICR), and OS. Individual patient data from the TALAPRO-2 trial and summary-level data from PROpel and MAGNITUDE trials were used for the relevant populations. The most recent data cutoffs available for each outcome were used. For TALAPRO-2 Cohorts 1 and 2, both rPFS and OS data were from the September 3, 2024 data cutoff. For PROpel, rPFS data were from the July 30, 2021 cutoff, which included BICR assessments not reported in subsequent data cuts; OS data were from the October 12, 2022 cutoff.^21^^,^^22^ For MAGNITUDE, both rPFS and OS data were based on the June 26, 2022 second interim analysis.^7^

### Selection of treatment effect modifiers

Unadjusted differences in treatment effect modifiers (TEMs) can bias comparative efficacy estimates. Twelve such TEMs were selected based on clinical relevance and evidence from the literature, and were ranked by an experienced clinician according to their expected impact on outcomes.^23^ Overall, the following variables were deemed to have strong effect-modifying potential (in ranked order): time to mCRPC from continuous ADT, presence of liver metastases, number of bone metastases, Eastern Cooperative Oncology Group (ECOG) performance status, Brief Pain Inventory-Short Form (BPI-SF) score, PSA kinetics or PSA level, Gleason score, hemoglobin level, lactate dehydrogenase (LDH) level, albumin level, alkaline phosphatase (ALP) level, and neutrophil-to-lymphocyte ratio (NLR). The availability of these TEMs across trials is shown in Table S.5. The primary analysis adjusted for all mutually available TEMs in each pairwise analysis. Additional scenario and exploratory analyses were conducted (Supplementary Appendix C, Supplementary Appendix D).

### Statistical analysis

#### Matching-adjusted indirect comparison

The methodology used in this analysis has been previously published.^14^ The following are additional details specific to the present analyses.

Due to the absence of a common comparator across the TALAPRO-2, PROpel, and MAGNITUDE trials, separate unanchored MAICs were conducted between TALA+ENZA and each comparator (OLAP+AAP and NIRA+AAP). Comparisons between TALA+ENZA and OLAP+AAP were performed in both the unselected and HRR-deficient populations, while comparisons between TALA+ENZA and NIRA+AAP were conducted in the HRR-deficient and *BRCA*m populations. Comparisons between TALA+ENZA and OLAP+AAP in the *BRCA*m population were not conducted due to cross-trial heterogeneity that could impact the relative efficacy estimates identified during the feasibility assessment phase (see “Results: Feasibility assessment” section below). All analyses were conducted using R version 4.3.2, following the methodological framework described by Signorovitch et al., 2012 and the NICE Decision Support Unit (DSU) Technical Support Document (TSD) 18.^24^^,^^25^

For each comparison, patients from TALAPRO-2 were separately assessed against the eligibility criteria of the comparator trial. For the comparison with PROpel, patients who did not meet PROpel’s eligibility criteria were excluded from the TALAPRO-2 dataset. Similarly, for the comparison with MAGNITUDE, TALAPRO-2 patients who did not meet MAGNITUDE’s eligibility criteria were excluded. For the HRR-deficient and *BRCA*m populations, patients from TALAPRO-2 with gene mutations that were not included or tested in PROpel or MAGNITUDE, respectively, were also excluded. The remaining TALAPRO-2 patients were then independently reweighted to match the distribution of ranked TEMs reported in PROpel or MAGNITUDE (Figure S.1). Population differences between TALAPRO-2 and each comparator trial were evaluated before and after adjustment using standardized mean differences (SMDs), where values between 0 and 0.1 indicated small differences, values greater than 0.1 and up to 0.2 indicated moderate differences, and values greater than 0.2 were considered substantial.^26^

Weights were generated using a generalized method-of-moments propensity score algorithm, applying inverse odds weighting to balance baseline characteristics between the TALAPRO-2 and comparator trial populations.^27^^,^^28^ The effective sample size (ESS) was calculated to reflect the sample size of the weighted population.^27^ Outcomes were reanalyzed using the weighted TALAPRO-2 dataset. rPFS and OS data were simulated for PROpel and MAGNITUDE from digitized published Kaplan–Meier (KM) curves using the Guyot method.^29^ Hazard ratios (HRs) and 95% confidence intervals (CIs) were estimated using weighted Cox proportional hazards models. Statistical significance was determined when the 95% CI did not include 1 and the *P*-value was less than .05. KM curves were plotted for the adjusted analyses. Since these analyses summarize relative efficacy using a Cox proportional hazards model, the plausibility of the proportional hazards assumption was assessed for each outcome and population of interest using the Grambsch-Therneau test, in accordance with NICE TSD 14 guidance.^30^^,^^31^

## Results

### Systematic literature review

Three pivotal Phase 3 RCTs evaluating the regimens of interest were identified from the SLR and included in the analysis: TALAPRO-2 (TALA+ENZA), PROpel (OLAP+AAP), and MAGNITUDE (NIRA+AAP) (Table 1). All three studies were assessed to be of good methodological quality according to the NICE STA Checklist, as shown in Table S.4.^20^

### Feasibility assessment

The feasibility assessment comparing TALAPRO-2 with PROpel and MAGNITUDE identified some differences between trials. All three were Phase 3, randomized, double-blind, multicenter trials evaluating a PARPi in combination with an ARPI. However, both comparator arms and treatment backbones differed: TALAPRO-2 assessed TALA+ENZA vs placebo plus ENZA, while PROpel and MAGNITUDE assessed OLAP+AAP and NIRA+AAP, respectively, vs placebo plus AAP. As a result, there was no shared comparator between TALAPRO-2 and either of the two trials due to different ARPI backbones (Supplementary Appendix F, Table S.7).

Trial eligibility criteria were broadly aligned in terms of age, ECOG performance status, histology, castration status, and bone metastases. Key differences included TALAPRO-2’s requirement for a BPI-SF item 3 score ≤3 and a life expectancy ≥12 months, compared to no pain score restriction and a ≥ 6-month life expectancy in PROpel (Table S.8). Consequently, 25.8% of patients in the OLAP+AAP arm of PROpel were symptomatic at baseline (BPI-SF item 3 score of ≥4 or opiate use) in the unselected population.^6^ TALAPRO-2 enrolled only patients who were treatment-naive in the mCRPC setting, whereas MAGNITUDE allowed ≤4 months of prior abiraterone acetate in mCRPC, with 23.6% of patients in the niraparib plus abiraterone acetate arm meeting this criterion.^7^ Prior ARPI and taxane-based chemotherapy in the castration-sensitive setting was permitted across all trials. In the HRR-deficient and *BRCA*m populations, differences in the inclusion of HRR gene alterations and the gene panels used to assess them were noted across the three trials (Supplementary Appendix E, Table S.6).

Baseline characteristics were generally comparable, though PROpel and MAGNITUDE had a higher proportion of Caucasian patients than TALAPRO-2 (Table 2). PROpel also had fewer patients with BPI-SF scores ≤3, since TALAPRO-2 excluded patients with BPI ≥4 (Table 2). Reporting of visceral metastases and radiographic progression at baseline varied, limiting assessment of comparability.

Outcome definitions were consistent across trials, with all trials using Response Evaluation Criteria in Solid Tumors version 1.1 (RECIST 1.1) and Prostate Cancer Working Group 3 (PCWG3) criteria. Radiographic progression-free survival assessed by BICR was used for all analyses to ensure comparability, despite PROpel’s primary endpoint being investigator-assessed rPFS.

Results for each of the feasibility components, including trial design, eligibility criteria, baseline patient characteristics, outcome availability and definitions, the nature of interventions comparators, and the availability of IPD, were reviewed and presented to a clinician experienced in treating patients with mCRPC. Based on this clinical assessment, the trials were considered sufficiently comparable to support unanchored MAICs in the following patient populations: TALA+ENZA vs OLAP+AAP in both the unselected and HRR-deficient populations, and TALA+ENZA vs NIRA+AAP in the HRR-deficient and *BRCA*m populations.

Several data-related concerns were identified that precluded comparative analyses between TALAPRO-2 (TALA+ENZA) and PROpel (OLAP+AAP) in the *BRCA*m population, including differences in follow-up duration, small sample size, and cross-over of KM curves, which indicate a violation of the proportional hazards assumption. A full explanation of these limitations is provided in Supplementary Appendix I, along with the balance table (Table S.13) and KM curves (Figures S.5 and S.6).

### MAIC results

#### TALA+ENZA vs OLAP+AAP: unselected population

##### Balance of populations

The unselected populations from TALAPRO-2 (*n* = 402) and PROpel (*n* = 399) were generally aligned in terms of eligibility criteria, with PROpel being broader in some respects (eg, BPI-SF); therefore, the full TALAPRO-2 IPD was retained for analysis.

Baseline characteristics before and after the adjustment process are presented in Table S.9. The analysis adjusted for three stratification factors, nine ranked TEMs, and two exploratory factors that were mutually available in TALAPRO-2 and PROpel. Baseline characteristics were generally similar between patients receiving TALA+ENZA in TALAPRO-2 and those receiving OLAP+AAP in PROpel. However, differences were observed in ECOG performance status, Gleason score, and ALP levels. TALAPRO-2 included a higher proportion of patients with ECOG performance status of 1 and Gleason score of ≥8, and elevated ALP levels, while PROpel had a greater proportion of patients with bone metastases. After reweighting, all mutually reported TEMs were balanced (SMD = 0), and the ESS for the TALA+ENZA arm was reduced by 25% in the primary analysis (ESS = 301) (Figure S.1).

##### MAIC analyses

**Figure 1.**
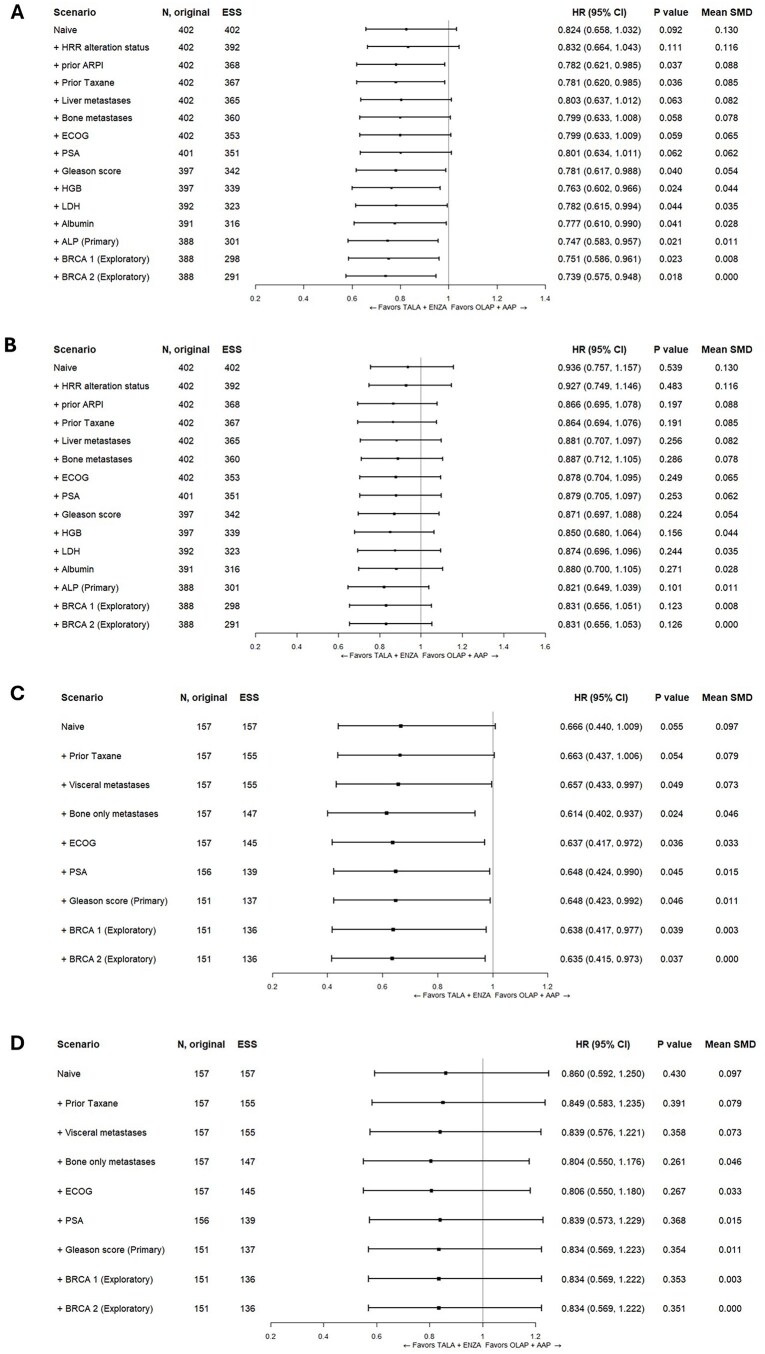
Forest Plots of HR (95% CIs) for TALA+ENZA vs OLAP+AAP. (A) rPFS (BICR) in the unselected population; (B) OS in the unselected population; (C) rPFS (BICR) in the HRR-deficient population; (D) OS in the HRR-deficient population. Results of the naive analysis and subsequent analyses which adjust for each new characteristic incrementally are shown. The primary analysis is indicated which adjusts for all factors listed above it. Patients with missing values for a given characteristic were excluded from the corresponding analysis. An HR below 1.0 indicates an improved outcome for TALA+ENZA relative to OLAP+AAP. Abbreviations: ALP, alkaline phosphatase level; ARPI, androgen receptor pathway inhibitor; BICR, blinded independent central review; CI, confidence interval; ECOG, Eastern Cooperative Oncology Group; ESS, effective sample size; HGB, hemoglobin level; HR, hazard ratio; HRR, homologous recombination repair; HRRm, homologous recombination repair biomarker positive; LDH, lactate dehydrogenase level; OLAP+AAP, olaparib plus abiraterone acetate; OS, overall survival; PSA, prostate specific antigen; rPFS, radiographic progression-free survival; SMD, standardized mean difference; TALA+ENZA, talazoparib plus enzalutamide.

**Figure 2.**
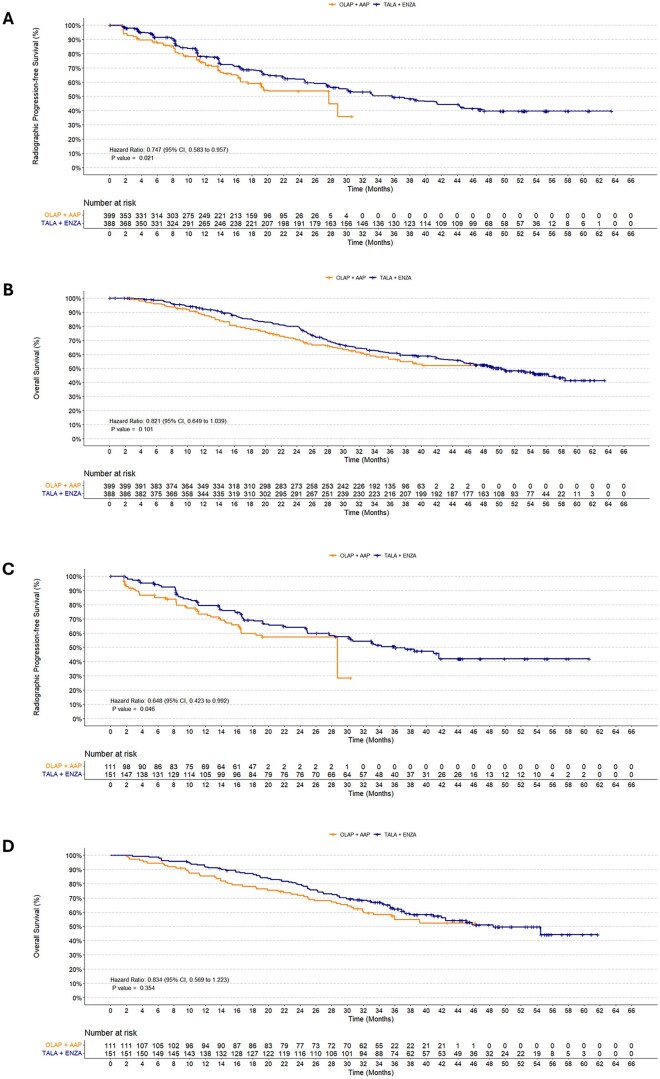
Kaplan–Meier Estimates for TALA+ENZA vs OLAP+AAP. (A) rPFS (BICR) in the unselected population; (B) OS in the unselected population; (C) rPFS (BICR) in the HRR-deficient population; (D) OS in the HRR-deficient population. Abbreviations: BICR, blinded independent central review; CI, confidence interval; HR, hazard ratio; HRR, homologous recombination repair; OLAP+AAP, olaparib plus abiraterone acetate; OS, overall survival; rPFS, radiographic progression-free survival; TALA+ENZA, talazoparib plus enzalutamide.

In the primary analysis of the unselected population, treatment with TALA+ENZA was associated with a statistically significant longer rPFS (HR: 0.747; 95% CI, 0.583 to 0.957; *P* = .021) than those receiving OLAP+AAP (Figures 1A and 2A). Consistent results were observed in the scenario and exploratory analyses (Figure 1A). Results for OS numerically favored TALA+ENZA compared to OLAP+AAP, but the difference was not statistically significant (HR: 0.821; 95% CI, 0.649 to 1.039; *P* = .101) (Figures 1B and 2B). Similar results for OS were observed in the scenario and exploratory analyses (Figure 1B).

#### TALA+ENZA vs OLAP+AAP: HRR-deficient population

##### Balance of populations

TALAPRO-2 Cohort 2 (*n* = 200) was matched to PROpel (*n* = 111) based on the presence of HRR gene alterations. Due to cross-trial differences in HRR gene assessment methods (Table S.6), 26 patients were removed from TALAPRO-2 Cohort 2 as a result of harboring HRR gene alterations not evaluated in PROpel. An additional 17 patients were excluded due to prior ARPI use in the castration-sensitive stage. While this variable was not reported for the HRR-deficient population in PROpel, only one patient in the unselected PROpel population received prior ARPI. To minimize differences between cohorts, these patients were removed, resulting in a final TALAPRO-2 sample size of 157. No additional exclusions were necessary, as the eligibility criteria in PROpel were otherwise similar or broader compared with TALAPRO-2.

Baseline characteristics before and after the adjustment process are presented in Table S.10. The analysis adjusted for one stratification factor, five ranked TEMs, and two exploratory factors that were mutually available in TALAPRO-2 Cohort 2 and PROpel. Following alignment on HRR gene status, baseline characteristics were generally comparable between the 157 patients receiving TALA+ENZA and the 111 patients receiving OLAP+AAP. Differences were observed in ECOG performance status, bone-only metastases, and PSA levels. Specifically, TALAPRO-2 Cohort 2 included a higher proportion of patients with ECOG performance status of 1, while PROpel had more patients with bone-only metastases and elevated PSA. After reweighting, all mutually reported TEMs were balanced (SMD = 0), and the resulting ESS for the TALA+ENZA arm was reduced by 13% in the primary analysis (ESS = 137) (Figure S.1).

### MAIC analyses

In the primary analysis of the HRR-deficient population, treatment with TALA+ENZA was associated with a statistically significant longer rPFS (HR: 0.648; 95% CI, 0.423 to 0.992; *P* = .046) than those receiving OLAP+AAP (Figures 1C and 2C). Scenario and exploratory analyses produced similar results (Figure 1C). Results for OS numerically favored TALA+ENZA over OLAP+AAP, but the difference was not statistically significant (HR: 0.834; 95% CI, 0.569 to 1.223; *P* = .354) (Figures 1D and 2D). Consistent OS results were observed across scenario and exploratory analyses (Figure 1D).

#### TALA+ENZA vs NIRA+AAP: HRR-deficient population

Full results comparing TALA+ENZA and NIRA+AAP in the HRR-deficient population are available in Supplementary Appendix H (Table S.12; Figures S.3 and S.4).

#### TALA+ENZA vs NIRA+AAP: *BRCA*m population

##### Balance of populations

TALAPRO-2 Cohort 2 (*n* = 71) was matched to MAGNITUDE (*n* = 113) based on the presence of *BRCA1, BRCA2, or BRCA* co-occurring with other HRR gene alterations tested for in MAGNITUDE (Table S.6). Two patients from TALAPRO-2 Cohort 2 were excluded due to co-occurring HRR alterations not assessed in MAGNITUDE, reducing the sample size to 69 patients. No further exclusions were necessary, as the key eligibility criteria in MAGNITUDE were either similar to or broader than those in TALAPRO-2 Cohort 2.

Baseline characteristics before and after the adjustment process are presented in Table S.11. The analysis adjusted for two stratification factors, seven ranked TEMs, and three exploratory factors that were mutually available in TALAPRO-2 Cohort 2 and MAGNITUDE and adjusted for in the analysis. Following alignment on HRR gene status, baseline characteristics were generally similar between the 69 patients receiving TALA+ENZA and the 113 patients receiving NIRA+AAP, with the exception of PSA and LDH levels, which were higher in MAGNITUDE. After reweighting, all mutually reported TEMs were balanced (SMD = 0), and the resulting ESS for the TALA+ENZA arm was reduced by 35% in the primary analysis (ESS = 45) (Figure S.1).

##### MAIC analyses

**Figure 3.**
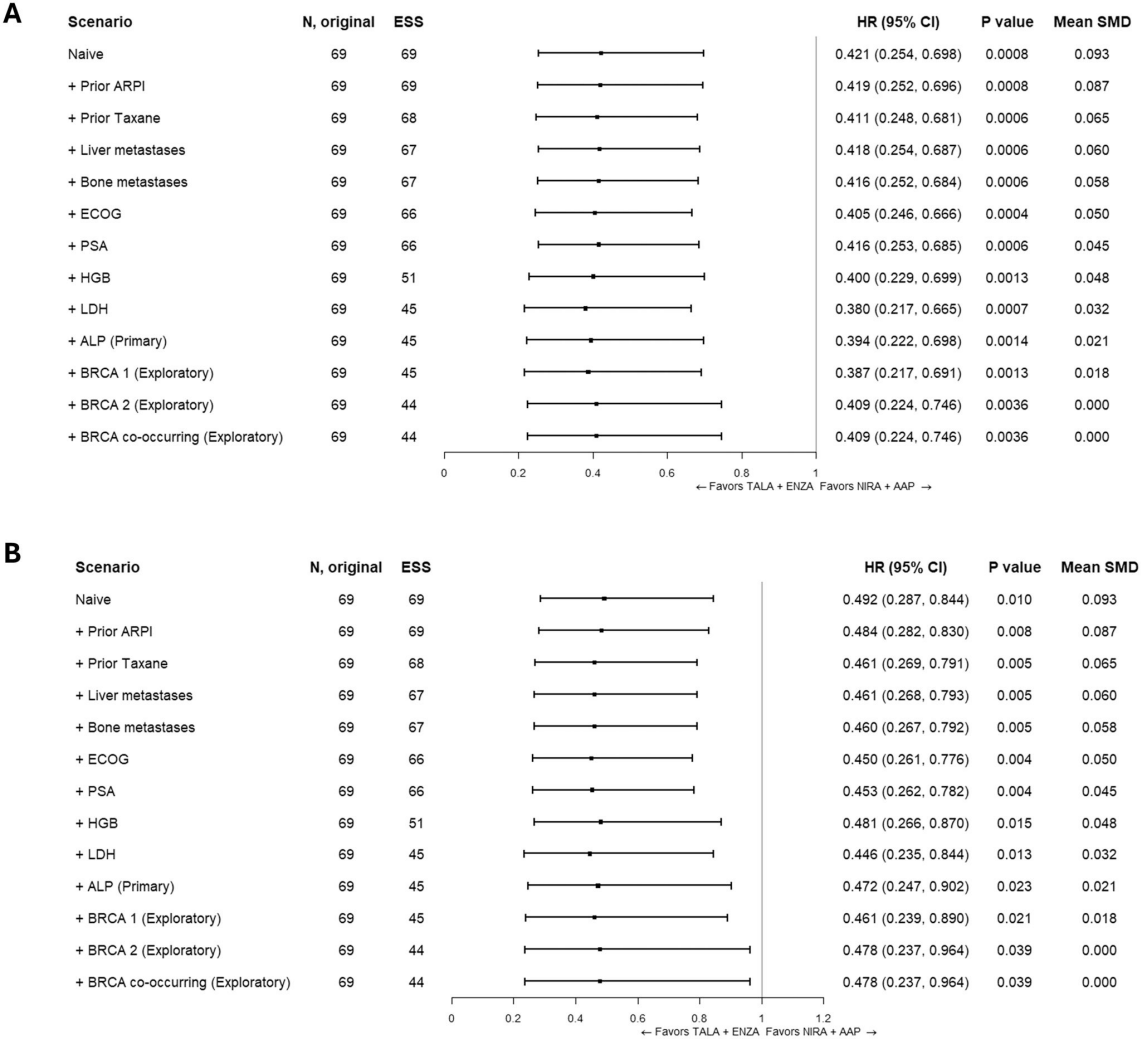
Forest Plots of HR (95% CIs) for TALA+ENZA vs NIRA+AAP. (A) rPFS (BICR) in the *BRCA*m population; (B) OS in the *BRCA*m population. Results of the naive analysis and subsequent analyses which adjust for each new characteristic incrementally are shown. The primary analysis is indicated which adjusts for all factors listed above it. Patients with missing values for a given characteristic were excluded from the corresponding analysis. An HR below 1.0 indicates an improved outcome for TALA+ENZA relative to NIRA+AAP. Abbreviations: ALP, alkaline phosphatase; ARPI, androgen receptor pathway inhibitor; BICR, blinded independent central review; *BRCA*m, *BRCA*-mutated; CI, confidence interval; ECOG, Eastern Cooperative Oncology Group; ESS, effective sample size; HGB, hemoglobin; HR, hazard ratio; LDH, lactate dehydrogenase; NIRA+AAP, niraparib plus abiraterone acetate; OS, overall survival; PSA, prostate specific antigen; rPFS, radiographic progression-free survival; SMD, standardized mean difference; TALA+ENZA, talazoparib plus enzalutamide.

**Figure 4.**
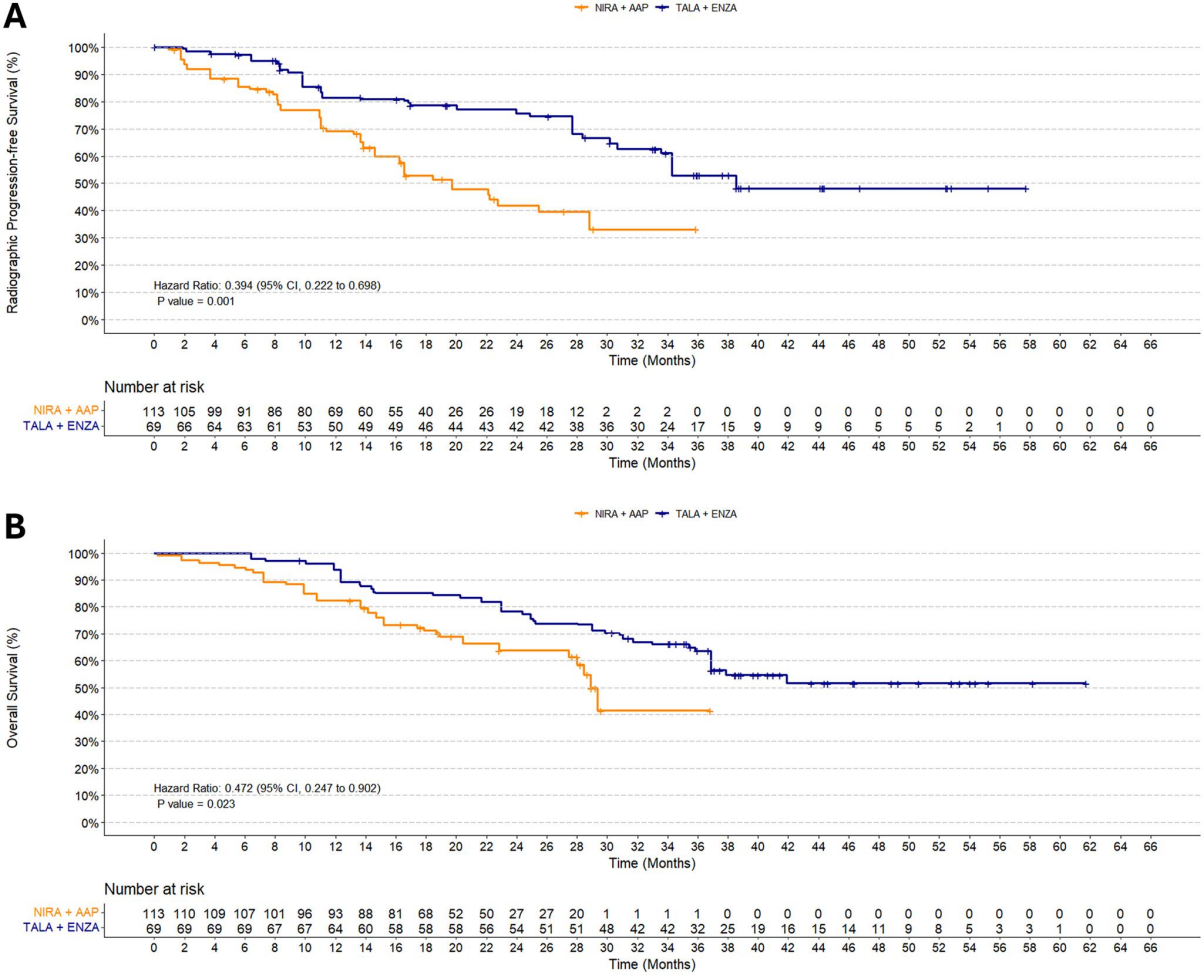
Kaplan–Meier Estimates for TALA+ENZA vs NIRA+AAP. (A) rPFS (BICR) in the *BRCA*m population; (B) OS in the *BRCA*m population. Abbreviations: BICR, blinded independent central review; *BRCA*m, *BRCA*-mutated; CI, confidence interval; HR, hazard ratio; NIRA+AAP, niraparib plus abiraterone acetate; OS, overall survival; rPFS, radiographic progression-free survival; TALA+ENZA, talazoparib plus enzalutamide.

Results of the primary analysis of the *BRCA*m population showed that treatment with TALA+ENZA was associated with a statistically significant longer rPFS (HR: 0.394; 95% CI, 0.222 to 0.698; *P* = .0014) (Figures 3A and 4A) and with a statistically significant lower risk of death (OS) (HR: 0.472; 95% CI, 0.247 to 0.902; *P* = .023) compared with NIRA+AAP (Figures 3B and 4B). Scenario and exploratory analyses for both endpoints yielded results that were consistent with the primary MAIC analyses (Figure 3A and B).

## Discussion

In the absence of head-to-head trials, the present study conducted unanchored MAICs to assess the relative efficacy of TALA+ENZA vs OLAP+AAP and vs NIRA+AAP across three clinically relevant populations: unselected, HRR-deficient, and *BRCA*m. The results showed that, TALA+ENZA was associated with a significantly lower risk of rPFS (as assessed by BICR) and death compared with OLAP+AAP in both the unselected and HRR-deficient populations (25% and 35% lower risk, respectively). No statistically significant differences in OS were observed between these treatments in either population. Comparisons with NIRA+AAP showed treatment with TALA+ENZA was associated with statistically significant improvements in both rPFS (as assessed by BICR) and OS in the HRR-deficient population (59% and 45% reduction in risk, respectively) and *BRCA*m population (61% and 53% reduction in risk, respectively). These findings suggest that TALA+ENZA may offer a more favorable efficacy profile across multiple patient populations and underscore the potential clinical value of this combination in first-line mCRPC.

To our knowledge, this is the first MAIC to compare first-line TALA+ENZA with OLAP+AAP and NIRA+AAP across multiple mCRPC populations. This analysis builds upon our previously published work that assessed TALA+ENZA vs OLAP+AAP in an unselected population and TALA+ENZA vs NIRA+AAP in the HRR-deficient population using the first TALAPRO-2 data cut.^14^ These analyses showed treatment with TALA+ENZA was associated with statistically significant improvements in rPFS and PSA response compared to OLAP+AAP in the unselected population, and in rPFS and time to PSA progression compared to NIRA+AAP in HRR-deficient patients, with similar efficacy across other endpoints.^14^ The current MAIC expands the scope by indirectly comparing these regimens in three distinct and clinically relevant mCRPC populations (unselected, HRR-deficient, and *BRCA*m) and incorporates the most mature and final data cutoffs for TALA+ENZA and the latest published data for comparator arms. Other ITCs in this space have primarily been network meta-analyses, which did not adjust for cross-trial differences in baseline characteristics and are therefore more susceptible to bias.^19^^,^^32–37^ In contrast, the current MAICs used IPD from TALAPRO-2 to reweight populations and adjust for key TEMs to improve the validity of comparative estimates. Where possible, primary endpoints were aligned across trials; for example, rPFS assessed by BICR was used consistently, despite PROpel’s primary endpoint being investigator-assessed rPFS, to better align patient populations and ensure comparability.

Several methodological strengths support the robustness of these findings. First, a comprehensive feasibility assessment was conducted to evaluate inter-trial heterogeneity. Furthermore, consistency in outcome definitions (eg, defined by RECIST 1.1 and PCWG3), assessment method (BICR vs investigator), and included gene alterations was ensured to facilitate fair comparisons and minimize bias. Second, the MAIC methodology followed established best practices.^24^^,^^25^ Third, the use of IPD from TALAPRO-2 enabled precise reweighting to align baseline characteristics with comparator trials, improving covariate balance and enhancing the credibility of comparative efficacy estimates. A recent feasibility assessment highlighted the difficulty of comparing PARPi combinations in the *BRCA*m subgroup due to limited published data, particularly for TALA+ENZA.^38^ However, the present analysis overcomes these limitations by leveraging updated IPD from TALAPRO-2, which includes detailed *BRCA*m population information and final OS results, enabling a more comprehensive and methodologically sound comparison with NIRA+AAP in the *BRCA*m population.

Nonetheless, several limitations must be acknowledged. First, unanchored MAICs rely on the assumption that all relevant TEMs are accounted for in the adjustment process. Despite efforts to identify and include key TEMs, residual confounding from unmeasured variables may persist. If patients in the TALA+ENZA arm of TALAPRO-2 had better unmeasured prognostic profiles than comparator cohorts, the MAIC could overestimate benefit; if they had worse unmeasured prognostic profiles, the estimates would bias against TALA+ENZA.The ability to adjust for TEMs was limited by the availability of these characteristics in the comparator trials. Second, variations in follow-up duration and the use of subsequent therapies post-discontinuation may impact OS estimates, which are particularly sensitive to post-progression treatment effects.^39^^,^^40^ Importantly, the inability to adjust for post-progression therapies should be considered when interpreting OS results, and therefore the OS findings should be viewed as exploratory. Third, while the TALAPRO-2 Cohort 2 (HRR-deficient population) and MAGNITUDE HRR-deficient populations were considered sufficiently similar to support unanchored MAICs, some differences in baseline characteristics were observed, as would be expected given slight differences between trials. To account for these differences, the present MAIC adjusted for a broad set of potential TEMs. Including these variables in the model was critical to ensure reliable estimates of comparative efficacy but led to a 70% reduction in ESS from the original sample size. Such reductions are not uncommon and have been reported in other oncology studies,^41^^,^^42^ and a literature review conducted by NICE found that ESS reductions ranged from 57% to 98% in published MAIC analyses that reported ESS.^24^ Importantly, although ESS reductions can decrease statistical precision, as reflected in the wider confidence intervals, the overall conclusion remained unchanged: TALA+ENZA was associated statistically significant benefit over NIRA+AAP in the HRR-deficient population for both survival outcomes across unadjusted and fully adjusted models. Thus, the ESS reduction should not diminish confidence in the findings but rather it is an expected consequence of appropriately adjusting for cross-trial variation in key prognostic factors. Moreover, the initially large sample size of TALAPRO-2 Cohort 2 ensured that this reduction in sample size did not preclude meaningful inference. Lastly, the broader inclusion criteria in PROpel (eg, BPI-SF scores) and prior abiraterone use in MAGNITUDE may have influenced patient characteristics and treatment outcomes in ways that could not be fully accounted for. Despite these differences, the trial populations were considered sufficiently similar to support the use of unanchored MAICs, based on input from a clinician experienced in treating patients with mCRPC (EC).

While efficacy is a key driver of treatment selection in mCRPC, other factors such as safety, tolerability, and quality of life also play critical roles in clinical decision-making. Although safety outcomes were considered for inclusion in this analysis, several methodological challenges limited the feasibility of conducting adjusted comparisons. Future comparative analyses that may be able to incorporate safety data and quality of life measures may provide a more comprehensive understanding of the clinical utility of these regimens. Additionally, as real-world evidence becomes available, complementary analyses may help validate these findings in real-world patient populations and further inform personalized treatment strategies. Therefore, the results of the current analysis should be interpreted alongside a review of the safety and quality of life profiles for each combination to support balanced clinical decision-making.

## Conclusion

This study provides indirect comparative evidence supporting the clinical benefit of TALA+ENZA over OLAP+AAP and NIRA+AAP in the first-line treatment of asymptomatic or mildly symptomatic mCRPC. Using IPD from TALAPRO-2 and a rigorous MAIC approach, TALA+ENZA showed consistent efficacy across unselected, HRR-deficient, and *BRCA*m populations. However, these findings should be interpreted in the context of the inherent limitations of unanchored indirect comparisons, which cannot establish definitive treatment superiority and remain subject to residual confounding and cross-trial differences. Nevertheless, in the absence of head-to-head trials, these results contribute useful comparative evidence that may help inform treatment decisions.

## Supplementary Material

oyag143_Supplementary_Data

## Data Availability

Data sharing not applicable to this article as no datasets were generated or analysed during the current study.

## References

[1] Powers E , Karachaliou GS, Kao C, et al Novel therapies are changing treatment paradigms in metastatic prostate cancer. J Hematol Oncol. 2020;13:144. 33115529 10.1186/s13045-020-00978-z PMC7594418

[2] Taylor AK , Kosoff D, Emamekhoo H, Lang JM, Kyriakopoulos CE. PARP inhibitors in metastatic prostate cancer. Front Oncol. 2023;13:1159557. 37168382 10.3389/fonc.2023.1159557 PMC10165068

[3] Calabrese M , Saporita I, Turco F, et al Synthetic lethality by co-inhibition of androgen receptor and polyadenosine diphosphate-ribose in metastatic prostate cancer. Int J Mol Sci. 2023;25:78. 38203248 10.3390/ijms25010078 PMC10779404

[4] Kostos L , Tran B, Azad AA. Combination of PARP inhibitors and androgen receptor pathway inhibitors in metastatic castration-resistant prostate cancer. Drugs. 2024;84:1093-1109. 39060912 10.1007/s40265-024-02071-y PMC11438617

[5] Agarwal N , Azad AA, Carles J, et al Talazoparib plus enzalutamide in men with first-line metastatic castration-resistant prostate cancer (TALAPRO-2): a randomised, placebo-controlled, phase 3 trial. Lancet. 2023;402:291-303. 37285865 10.1016/S0140-6736(23)01055-3

[6] Saad F , Clarke NW, Oya M, et al Olaparib plus abiraterone versus placebo plus abiraterone in metastatic castration-resistant prostate cancer (PROpel): final prespecified overall survival results of a randomised, double-blind, phase 3 trial. Lancet Oncol. 2023;24:1094-1108. 37714168 10.1016/S1470-2045(23)00382-0

[7] Chi K , Sandhu S, Smith M, et al Niraparib plus abiraterone acetate with prednisone in patients with metastatic castration-resistant prostate cancer and homologous recombination repair gene alterations: second interim analysis of the randomized phase III MAGNITUDE trial. Ann Oncol. 2023;34:772-782. 37399894 10.1016/j.annonc.2023.06.009 PMC10849465

[8] EMA. Talzenna: talazoparib. Updated July 18, 2025. Accessed August 19, 2025, https://www.ema.europa.eu/en/medicines/human/EPAR/talzenna#overview

[9] FDA. FDA approves talazoparib with enzalutamide for HRR gene-mutated metastatic castration-resistant prostate cancer. Updated June 20, 2023. Accessed August 18, 2025, https://www.fda.gov/drugs/drug-approvals-and-databases/fda-approves-talazoparib-enzalutamide-hrr-gene-mutated-metastatic-castration-resistant-prostate

[10] EMA. Lynparza: olaparib. Updated July 31, 2025. Accessed August 19, 2025, https://www.ema.europa.eu/en/medicines/human/EPAR/lynparza

[11] FDA. FDA approves olaparib with abiraterone and prednisone (or prednisolone) for BRCA-mutated metastatic castration-resistant prostate cancer. Updated May 31, 2023. Accessed August 19, 2025, https://www.fda.gov/drugs/drug-approvals-and-databases/fda-approves-olaparib-abiraterone-and-prednisone-or-prednisolone-brca-mutated-metastatic-castration

[12] EMA. Akeega: niraparib/ abiraterone acetate. Updated August 19, 2024. Accessed August 19, 2025, https://www.ema.europa.eu/en/medicines/human/EPAR/akeega

[13] FDA. FDA approves niraparib and abiraterone acetate plus prednisone for BRCA-mutated metastatic castration-resistant prostate cancer. Updated August 11, 2023. Accessed August 19, 2025, https://www.fda.gov/drugs/resources-information-approved-drugs/fda-approves-niraparib-and-abiraterone-acetate-plus-prednisone-brca-mutated-metastatic-castration

[14] Castro E , Wang D, Walsh S, et al Talazoparib plus enzalutamide versus olaparib plus abiraterone acetate and niraparib plus abiraterone acetate for metastatic castration-resistant prostate cancer: a matching-adjusted indirect comparison. Prostate Cancer Prostatic Dis. 2025;28:817-827. 39645562 10.1038/s41391-024-00924-x PMC12399417

[15] Moher D , Shamseer L, Clarke M, et al PRISMA-P Group. Preferred reporting items for systematic review and meta-analysis protocols (PRISMA-P) 2015 statement. Syst Rev. 2015;4:1. 25554246 10.1186/2046-4053-4-1 PMC4320440

[16] Page MJ , McKenzie JE, Bossuyt PM, et al The PRISMA 2020 statement: an updated guideline for reporting systematic reviews. BMJ. 2021;372:n71. 33782057 10.1136/bmj.n71 PMC8005924

[17] Page MJ , McKenzie JE, Bossuyt PM, et al Updating guidance for reporting systematic reviews: development of the PRISMA 2020 statement. J Clin Epidemiol. 2021;134:103-112. 33577987 10.1016/j.jclinepi.2021.02.003

[18] Shamseer L , Moher D, Clarke M, et al PRISMA-P Group Preferred reporting items for systematic review and meta-analysis protocols (PRISMA-P) 2015: elaboration and explanation. BMJ. 2015;350:g7647. 25555855 10.1136/bmj.g7647

[19] Castro E , Ellis J, Craigie S, et al Comparative efficacy and safety of talazoparib plus enzalutamide and other first-line treatments for metastatic castration-resistant prostate cancer. Oncologist. 2025;30:oyae237. 39427229 10.1093/oncolo/oyae237 PMC11954501

[20] Nice. Critical appraisal of the relevant clinical effectiveness evidence. Updated December 3, 2024. Accessed August 19, 2025, https://www.nice.org.uk/process/pmg24/chapter/clinical-effectiveness#quality-assessment-of-the-relevant-clinical-effectiveness-evidence

[21] Clarke NW , Armstrong AJ, Thiery-Vuillemin A, et al Abiraterone and olaparib for metastatic castration-resistant prostate cancer. NEJM Evid. 2022;1:EVIDoa2200043. 10.1056/EVIDoa2200043 38319800

[22] ODAC. Meeting of the Oncologic Drugs Advisory Committee - AstraZeneca Briefing Document. Updated April 28, 2023. Accessed August 19, 2025, https://www.fda.gov/advisory-committees/advisory-committee-calendar/april-28-2023-meeting-oncologic-drugs-advisory-committee-meeting-announcement-04282023#event-materials

[23] Armstrong AJ , Lin P, Higano CS, et al Development and validation of a prognostic model for overall survival in chemotherapy-naïve men with metastatic castration-resistant prostate cancer. Ann Oncol. 2018;29:2200-2207. 30202945 10.1093/annonc/mdy406 PMC6888025

[24] Phillippo D , Ades T, Dias S, Palmer S, Abrams KR, Welton N. NICE DSU technical support document 18: methods for population-adjusted indirect comparisons in submissions to NICE. 2016.

[25] Signorovitch JE , Sikirica V, Erder MH, et al Matching-adjusted indirect comparisons: a new tool for timely comparative effectiveness research. Value Health. 2012;15:940-947. 22999145 10.1016/j.jval.2012.05.004

[26] Austin PC. Balance diagnostics for comparing the distribution of baseline covariates between treatment groups in propensity‐score matched samples. Stat Med. 2009;28:3083-3107. 19757444 10.1002/sim.3697 PMC3472075

[27] Phillippo DM , Ades AE, Dias S, Palmer S, Abrams KR, Welton NJ. Methods for population-adjusted indirect comparisons in health technology appraisal. Med Decis Making. 2018;38:200-211. 28823204 10.1177/0272989X17725740 PMC5774635

[28] Signorovitch JE , Wu EQ, Yu AP, et al Comparative effectiveness without head-to-head trials: a method for matching-adjusted indirect comparisons applied to psoriasis treatment with adalimumab or etanercept. Pharmacoeconomics. 2010;28:935-945. 20831302 10.2165/11538370-000000000-00000

[29] Guyot P , Ades A, Ouwens MJ, Welton NJ. Enhanced secondary analysis of survival data: reconstructing the data from published Kaplan–Meier survival curves. BMC Med Res Methodol. 2012;12:9. 22297116 10.1186/1471-2288-12-9 PMC3313891

[30] Grambsch PM , Therneau TM. Proportional hazards tests and diagnostics based on weighted residuals. Biometrika. 1994;81:515-526.

[31] Latimer N. NICE DSU technical support document 14: survival analysis for economic evaluations alongside clinical trials-extrapolation with patient-level data. Report by the Decision Support Unit. 2011:2.

[32] Ai J , Jian L, Wen X, et al Comparative effectiveness of first-line systemic treatments for metastatic castration-resistant prostate cancer: a systematic review and network meta-analysis. Clin Transl Oncol. 2024;26:2559-2571. 38750344 10.1007/s12094-024-03506-4

[33] Chen X , Pan Y, Wang Q, et al Comparative efficacy of olaparib in combination with or without novel antiandrogens for treating metastatic castration-resistant prostate cancer. Front Endocrinol (Lausanne). 2023;14:1225033. 38027160 10.3389/fendo.2023.1225033 PMC10644304

[34] Huang Y , He H, Liang L, et al Efficacy and safety of PARP inhibitors in the treatment of prostatic cancer: a systematic review and network meta-analysis. Chin Clin Oncol. 2024;13:64-64. 39238347 10.21037/cco-24-82

[35] Liu Y , Deng X, Wen Z, et al Comparing efficacy of first-line treatment of metastatic castration resistant prostate cancer: a network meta-analysis of randomized controlled trials. Front Pharmacol. 2023;14:1290990. 38074136 10.3389/fphar.2023.1290990 PMC10702556

[36] McCool R , Fleetwood K, Glanville J, Arber M, Goodall H, Naidoo S. Systematic review and network meta-analysis of treatments for chemotherapy-naive patients with asymptomatic/mildly symptomatic metastatic castration-resistant prostate cancer. Value Health. 2018;21:1259-1268. 30314628 10.1016/j.jval.2018.03.012

[37] Zhang D , Weng H, Zhu Z, Gong W, Ma Y. Evaluating first-line therapeutic strategies for metastatic castration-resistant prostate cancer: a comprehensive network meta-analysis and systematic review. Front Oncol. 2024;14:1378993. 38686197 10.3389/fonc.2024.1378993 PMC11056588

[38] De Santis M , Breijo SM, Robinson P, et al Feasibility of indirect treatment comparisons between niraparib plus abiraterone acetate and other first-line poly ADP-Ribose polymerase inhibitor treatment regimens for patients with BRCA1/2 mutation-positive metastatic castration-resistant prostate cancer. Adv Ther. 2024;41:3039-3058. 38958846 10.1007/s12325-024-02918-6 PMC11263413

[39] FDA. Clinical Trial Endpoints for the Approval of Cancer Drugs and Biologics: Guidance for Industry. Updated May 7, 2020. Accessed August 19, 2025, https://www.fda.gov/regulatory-information/search-fda-guidance-documents/clinical-trial-endpoints-approval-cancer-drugs-and-biologics

[40] He P , Ma H, Lu CC, et al Ensuring quality and interpretability of progression free survival and overall survival in oncology clinical trials. Ther Innov Regul Sci. 2025;59:1495-1505. 40762767 10.1007/s43441-025-00848-1

[41] Armstrong AJ , Pandya BJ, Bhadauria HS, et al Matching-adjusted indirect comparison of enzalutamide versus darolutamide doublet in mHSPC. Future Oncol. 2025;21:2459-2469. 40654300 10.1080/14796694.2025.2526324 PMC12330274

[42] Atallah E , Mauro MJ, Hochhaus A, et al Matching-adjusted indirect comparison of asciminib versus other treatments in chronic-phase chronic myeloid leukemia after failure of two prior tyrosine kinase inhibitors. J Cancer Res Clin Oncol. 2023;149:6247-6262. 36707445 10.1007/s00432-022-04562-5 PMC10356870

[43] Fizazi K , Azad AA, Matsubara N, et al Talazoparib plus enzalutamide in men with HRR-deficient metastatic castration-resistant prostate cancer: final overall survival results from the randomised, placebo-controlled, phase 3 TALAPRO-2 trial. Lancet. 2025;406:461-474. 40683287 10.1016/S0140-6736(25)00683-X

[44] Chi KN , Rathkopf D, Smith MR, et al MAGNITUDE Principal Investigators. Niraparib and abiraterone acetate for metastatic castration-resistant prostate cancer. J Clin Oncol. 2023;41:3339-3351. 36952634 10.1200/JCO.22.01649 PMC10431499

