# Supplementary figures and images for: Matching-adjusted indirect comparisons of PARP inhibitor combinations in metastatic castration-resistant prostate cancer across key populations
Source: Oncologist. 2026 Apr 16;31(5):oyag143. doi: 10.1093/oncolo/oyag143 (PMC13123856; doi:10.1093/oncolo/oyag143)

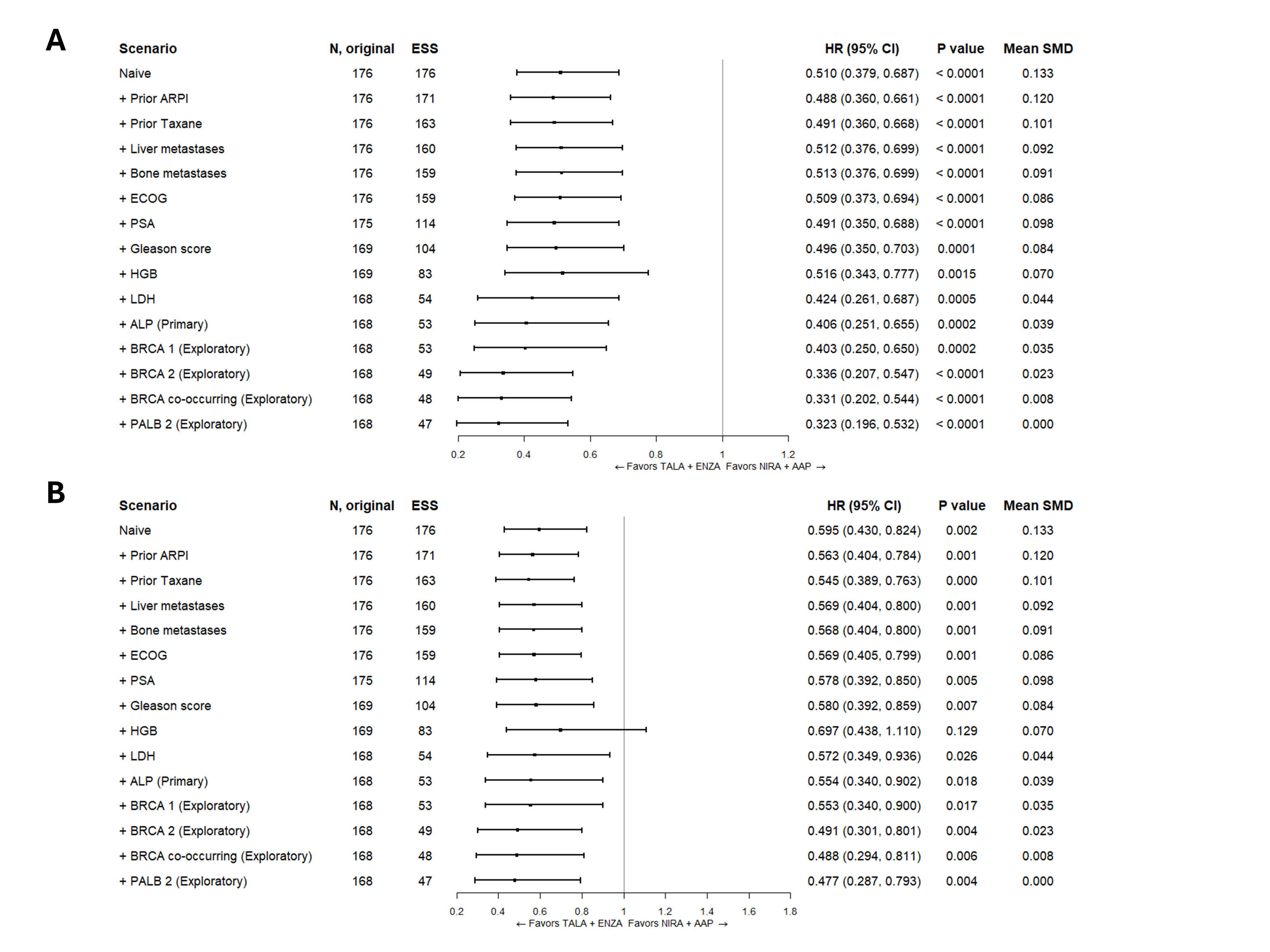

Supplement: oyag143_Supplementary_Data [file oyag143_supplementary_data.zip › Fig S.3.tif]

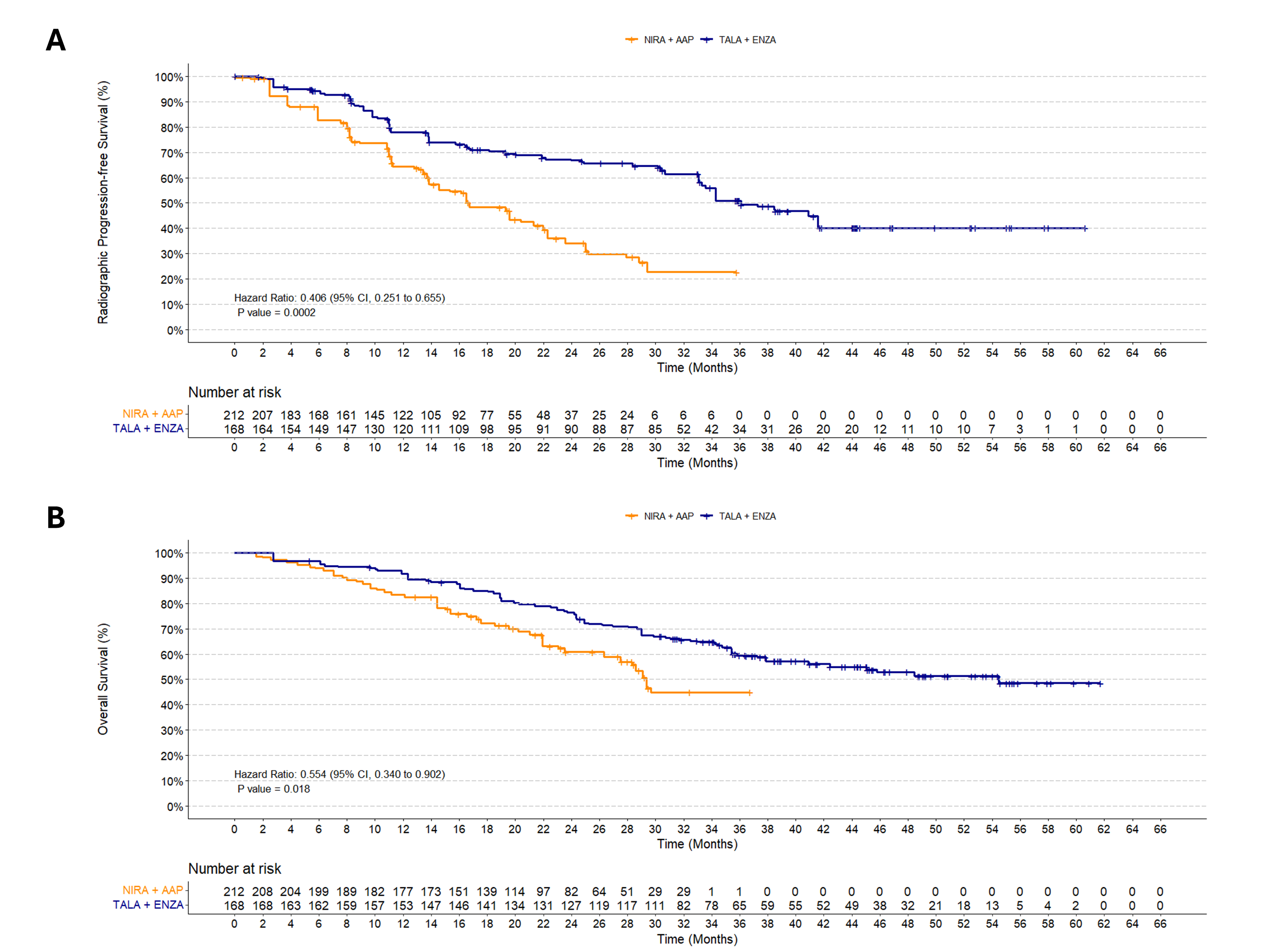

Supplement: oyag143_Supplementary_Data [file oyag143_supplementary_data.zip › Fig S.4.tif]

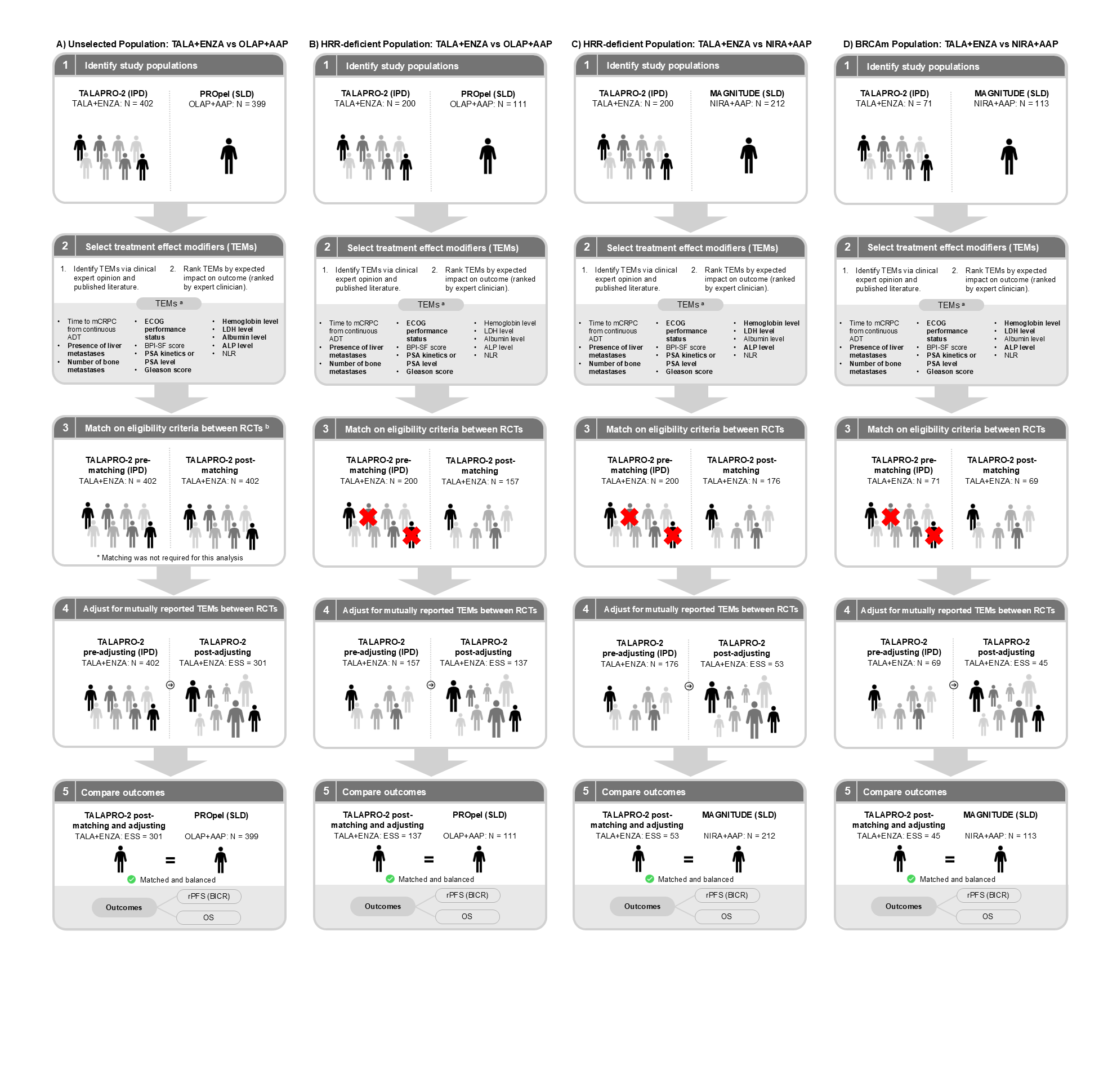

Supplement: oyag143_Supplementary_Data [file oyag143_supplementary_data.zip › Figure S.1.tif]

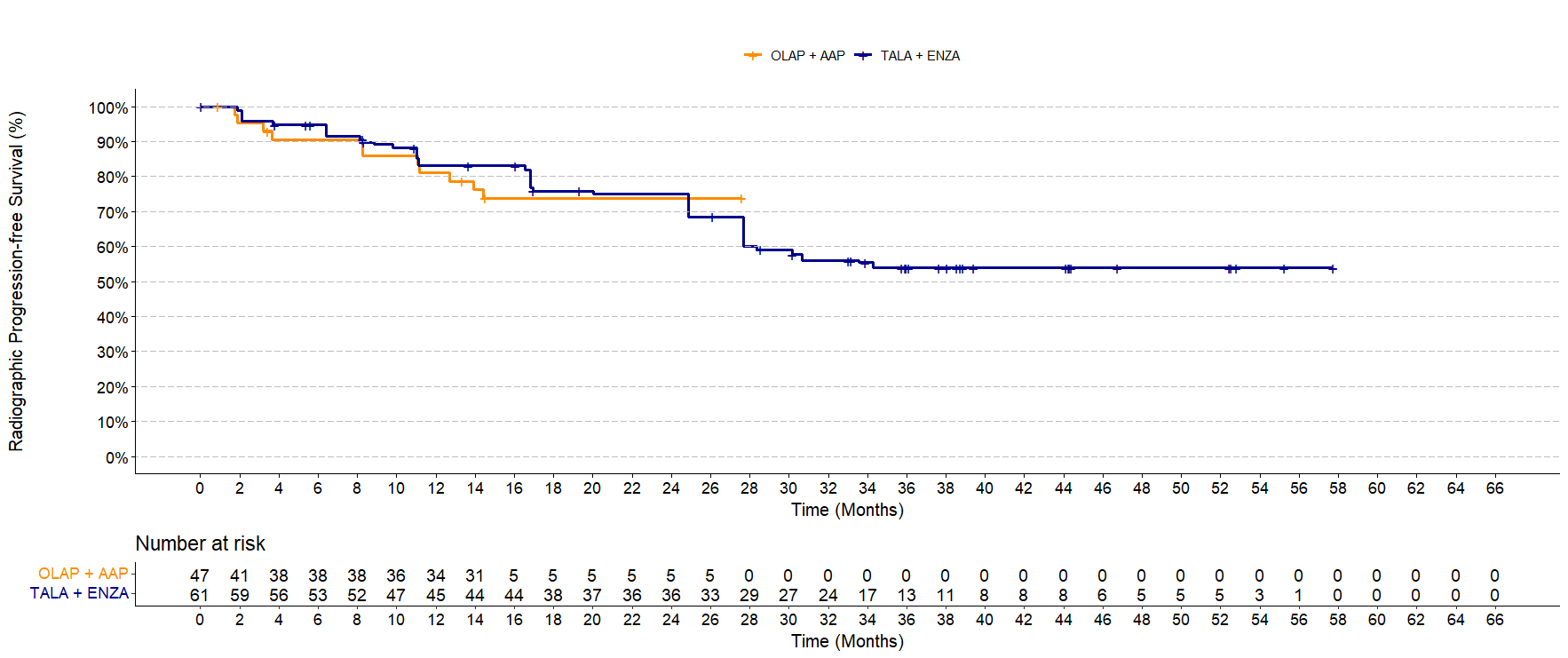

Supplement: oyag143_Supplementary_Data [file oyag143_supplementary_data.zip › Figure S.5.tiff]

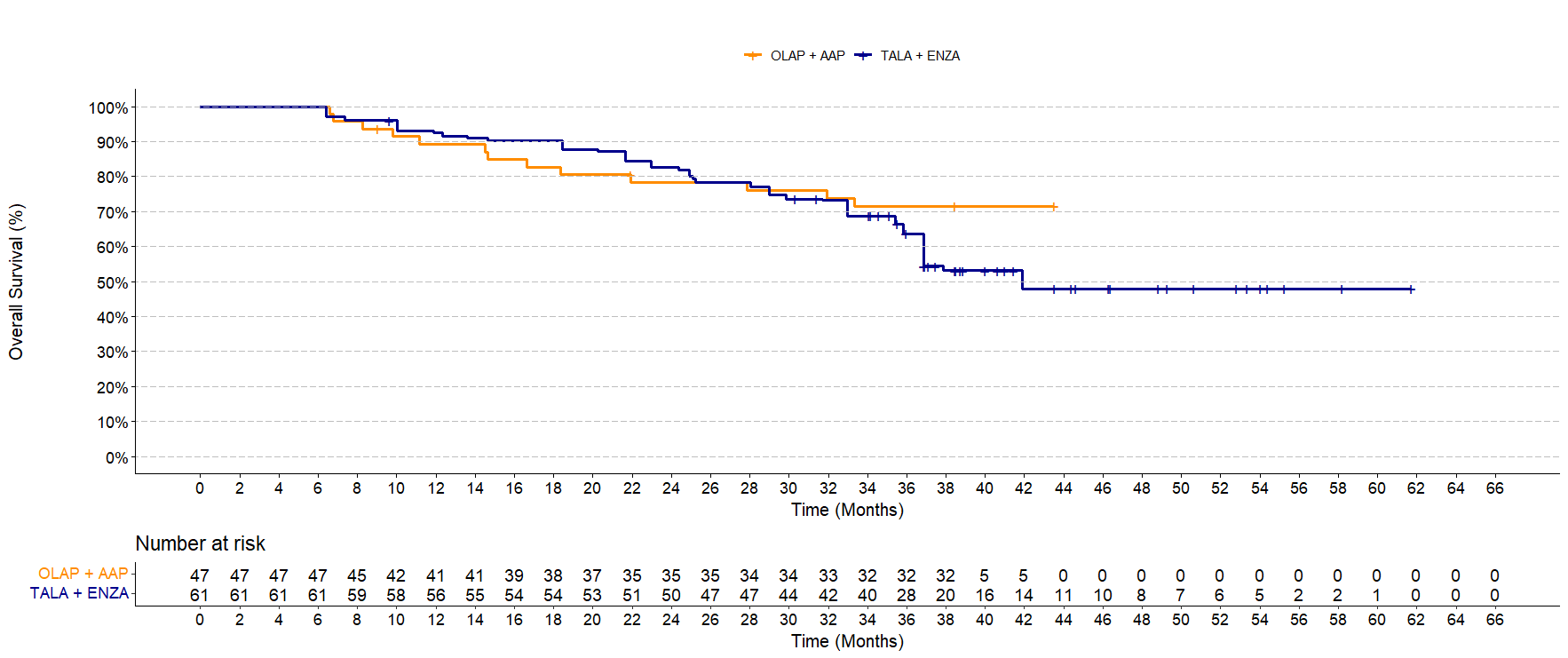

Supplement: oyag143_Supplementary_Data [file oyag143_supplementary_data.zip › Figure S.6.tiff]
